# The right to terminate pregnancy (abortion): reflections from Turkey

**DOI:** 10.1093/jlb/lsad023

**Published:** 2023-09-03

**Authors:** Hatice Kübra Ercoşkun Şenol, Pelin Ercoşkun

**Affiliations:** Faculty of Law, Atatürk University, Erzurum 25240, Turkey; Faculty of Medicine, Istanbul University—Cerrahpaşa, Istanbul 34098, Turkey

**Keywords:** abortion, embryo, fetus, termination of pregnancy, viability

## Abstract

In this article, the right to terminate pregnancy is discussed comparatively in terms of the situation in Turkey and the rest of the world. We have concluded that certain minimum conditions must be met to be able to talk about the full recognition of this right. Recognizing that pregnancy can be terminated voluntarily is the most important of these conditions. Just as the period in which this right can be exercised should not be kept short, it should also be accepted that it can only be used based on the will of a pregnant person who has the power of discernment. In addition, certain obligations must be avoided, such as receiving counseling services before the termination of pregnancy and then waiting for a certain period of time to pass. In cases where pregnancy can be terminated because of an anomaly, a disease, or a crime, it is important to make clear arrangements that will not make practitioners, especially physicians, hesitate to perform such a procedure. Finally, the State must never forget that it is obliged to organize services for the termination of pregnancies in a way that is easily accessible to everyone who may need this procedure.

## I. INTRODUCTION

In this article, the right to terminate pregnancy and the legal regulations in Turkish law regarding this right are discussed. The actual obstacles to this right are also evaluated. Therefore, two main problems are discussed in this article. The first is whether the regulations regarding the right to terminate pregnancy in Turkish law are accurate and sufficient.

In Turkish law, it is accepted that an embryo/fetus has no legal personality. As a matter of fact, according to the Turkish Civil Code (TCC), personality begins with full^1^ and right^2^ birth^3^ and ends with death (Art. 28, Para. 1). A person acquires the capacity to have rights and obligations from the moment of conception, subject to the fulfillment of the delaying condition^4^ of being born full and alive (Art. 28, Para. 2).^5^ Thus, an embryo/fetus, which has the capacity to have rights from the moment of conception, provided that it is born full and alive in the future and which does not yet have a personality, must be considered as part of the pregnant person.^6^

Although the embryo/fetus that does not have a personality is considered part of the pregnant person, in Turkish law, the right of the fetus to a future life is protected to a certain extent by not accepting the idea that the pregnant person can terminate her pregnancy as she wishes within the framework of her right to self-determination and disposition on her own body.^7^ Legal regulations on this issue are set out in the Turkish Penal Code (TPC), the Population Planning Law (PPL), and the Regulation on the Execution and Supervision of Uterine Evacuation and Sterilization Services (hereinafter referred to as ‘Regulation’). These regulations will be analyzed in this article in light of comparative law, doctrinal opinions, and judicial decisions.

The second problem to be addressed in this article is the actual obstacles to the right to terminate pregnancy in Turkey. As is known, the legal acceptance of this right does not make any sense on its own. For this right to fully come into existence, its exercise should not be hindered. In particular, it is very important that services for the termination of pregnancy are accessible.^8^ In this context, the actual obstacles to the use of such a right will be determined in this article, and solutions that will contribute to the accessibility of the right will be presented.

## II. LEGAL REGULATIONS ON THE TERMINATION OF PREGNANCY

### II.A. Voluntary Termination of Pregnancy (PPL Art. 5, Para. 1)

#### II.A.1. In General

According to the PPL, until the end of the 10th week of pregnancy, a uterus may be evacuated upon request if there is no medical inconvenience to the mother’s health (Art. 5, Para. 1). Under this provision, one of the conditions for the voluntary termination of pregnancy is that this procedure will not cause any harm to the health of a pregnant person requesting termination. Another condition is that the 10th week of pregnancy has not yet expired.

In our opinion, the period adopted in this provision is short. While some other legal systems that accept the right to terminate pregnancy on request have also adopted shorter periods, such as 10, 9, or even 8 weeks, the vast majority of legal systems in this category have adopted periods exceeding 10 weeks. In fact, a period of 12 weeks is most commonly adopted.^9^ Thus, if it is accepted that pregnancy can be terminated on an optional basis in Turkey, it would be accurate to extend the period to at least 12 weeks so that this right can be used as required.^10^ Indeed, in calculations based on the first day of the last menstrual period, pregnancy usually cannot be understood in the first 4 or 5 weeks. Once a pregnancy is detected, time may be needed to make a decision on the continuation, or termination, of a pregnancy and/or to have opportunities to terminate it. However, waiting for this period to be kept too long can be unrealistic, at least for now. The notion that pregnancy can be terminated on an optional basis, especially in advanced gestation periods, when an embryo/fetus reaches the ability to live independently of a pregnant person (viability), will be met with great resistance by part of Turkish society. As a matter of fact, one of the most important factors determining the perspectives of individuals forming society’s opinions toward the termination of pregnancy is their religious beliefs.^11^ Among the Hanafi Sect, which comprises the majority in Turkey,^12^ there are fatwas where an embryo/fetus should be considered a person from the moment of falling into a womb. The High Board of Religious Affairs of the Presidency of Religious Affairs, based on these fatwas, made explanations in this direction.^13^ Another fatwa in the Hanafi Sect states that pregnancy can be terminated from the moment an embryo/fetus falls into a womb until the 120th day when a soul is given to an embryo/fetus.^14^

In some legal systems that accept the right of voluntary termination of pregnancy, the person requesting the termination is obliged to receive consultancy services, after which a certain waiting period is expected to expire and the pregnancy is terminated if there is no change in the requester’s decision at the end of this period.^15^ It is suggested that such arrangements should be made in Turkey because of the physical and mental trauma that such termination can cause in a person.^16^ In our opinion, because the necessary information is provided by a physician before a pregnancy is terminated, it is not necessary to require additional counseling services. There is also the risk that this service will be used as a means to pressure a person not to terminate her pregnancy.^17^ In some legal systems that include the obligation to receive consultancy services, there is a corresponding responsibility to make detailed explanations about the risks of termination and the development of an embryo/fetus while this service is provided.^18^ This situation shows that the obligation to receive counseling services may actually be used to discourage persons from terminating their pregnancies.^19^ Furthermore, it would not be currently appropriate to also include a short waiting period of 10 weeks. After the extension of the 10-week period comes to the agenda, the positive and negative consequences of introducing such a waiting period can be discussed. However, it should not be overlooked that studies^20^ have revealed that most of those who want to terminate their pregnancies are not satisfied with the waiting period. In fact, this obligation increases the cost of the termination of pregnancy, as it requires one more trip to be made on the part of the individual making the request.

#### II.A.2. Persons Requiring Consent to Terminate a Pregnancy

Certainly, the first person to consent to the termination of a pregnancy is the pregnant person (PPL Art. 6, Para. 1, Sentence 1). Aside from exceptional circumstances, it is not possible to initiate such termination against the consent of a pregnant person. However, if a pregnant person is constantly deprived of the power of discernment, in other words, if she is completely incapable, it is clear that her consent for the termination is meaningless. This issue is stated in the PPL as ‘…her consent is not sought for uterine evacuation for a pregnant woman who does not have freedom of consciousness due to mental disability’ (Art. 6, Para. 1, Sentence 2). To terminate the pregnancy of a person in this situation, permission must be obtained from a guardian if she is under custody or from the magistrate, together with her guardian, if she is under guardianship.^21^

If a person who has the power of discernment is not yet an adult or has certain limitations, she must first give her consent to terminate her pregnancy. In this situation, initiating a termination of pregnancy also requires the permission of her guardian if she is under custody, and that of the magistrate together with her guardian if she is under guardianship (PPL Art. 6, Para. 1, Sentence 1). For this reason, although this person wants the pregnancy to be terminated, it cannot be done if her legal representative and/or the magistrate does not allow it. However, from the general provision that does not seek the consent of a legal representative in exercising the rights strictly attached to a person (TCC Art. 16, Para. 1, Sentence 2), it would be more accurate to accept that only a pregnant person’s will should be acted upon in terms of the right to terminate pregnancy, which is a right of this nature.^22^

It is also clear that if some person’s pregnancy or their desire to terminate it is reported to their legal representatives, they will be at risk of violence, especially stigmatization. The importance of the pregnant person having the right to terminate her pregnancy of her own free will is also emphasized in the Abortion Care Guideline published by the World Health Organization (WHO) in 2022.^23^

Although we think that it is not true, if the person who wants to terminate her pregnancy because of the current legal regulation is limitedly incapable, she has to obtain permission from her parent, guardian, or, according to the situation, from the magistrate.^24^ However, there is no provision in the PPL regarding how to act in cases where there is no legal representative or where it is not possible to obtain permission from the current legal representative, for example, because this person’s whereabouts are unknown. Thus, this gap should be filled with a rule stating that permission from the legal representative will no longer be required in such cases.^25^

If the pregnant person is married, the PPL seeks the consent of her husband to terminate the pregnancy within a period that does not exceed 10 weeks (Art. 6, Para. 2). Because of this regulation, the Turkish legal system is one of the few legal systems in the world that seeks the consent of the spouse in the termination of pregnancy.^26^ The Civil Code (repealed Turkish Law Civil) that was in force at the time the PPL came into force resolved that the husband was the head of the household and that the livelihood of his wife and children belonged to him (Art. 152). However, this provision, which constituted a legal example of discrimination based on gender, was not included in the new Civil Code (TCC). In the TCC, the requirement that the spouses must jointly manage the marriage union (Art. 186, Para. 2) and are obliged to oversee the care, education, and supervision of their children together (Art. 185, Para. 2) has been adjudicated. In light of these changes made in the Civil Code, the provision of the PPL, which seeks the consent of the spouse to terminate the pregnancy of a married person, should also be disposed of.^27^ In addition, although a paternity relationship will be established between the child and the father, as between the child and the mother,^28^ the continuation or termination of pregnancy is an event that has a greater effect on the pregnant person, as either option occurs directly on the pregnant person’s body.

Hence, in cases in which spouses do not agree with the termination of pregnancy, it would be better to act according to the will of the pregnant person. On the same grounds, the European Court of Human Rights (ECtHR) has ruled that legal regulations that do not allow a father to have a say in the termination of pregnancy do not constitute a violation of his right to privacy under Article 8 of the European Convention on Human Rights.^29^ In addition, the provision requiring the consent of a spouse for termination creates discrimination between married and unmarried pregnant people. It is also clear that an individual may be at risk of violence or stigmatization, because her pregnancy or desire to terminate it is reported to her spouse.^30^ The importance of the right to terminate a pregnancy in accordance with one’s own volition has also been emphasized in the Abortion Care Guideline published by the WHO in 2022.^31^ In the 2016 report on Turkey by the United Nations Committee on the Elimination of Discrimination Against Women, it was recommended that termination of pregnancy should be based solely on the decision of the pregnant person.^32^

Although we believe this is not right, within the framework of the current legal regulation, if a person who wants to terminate her pregnancy is married, she must obtain the consent of her spouse to do so.^33^ However, the PPL does not include a provision on how to act in cases where it is not possible to obtain consent from a spouse, such as when the whereabouts of a spouse are unknown. Thus, this gap should be filled with a rule stating that, in such cases, it is no longer necessary to obtain permission from a spouse.

For spouses who cannot agree to the termination of pregnancy, as they have already disagreed on an important issue regarding the marriage union, they may request the intervention of a judge individually or together in accordance with the TCC Art. 195, Para. 1. In this case, the judge will attempt to reconcile the spouses and may request the assistance of experts with the mutual consent of both parties. However, judges and experts who intervene in such a dispute and try to reconcile spouses should pay attention to the fact that the continuation or termination of pregnancy is an event that has a greater effect on the pregnant person. Moreover, a judge’s role here is to try to reconcile the spouses; otherwise, it is not possible for the judge to decide whether a pregnancy can be terminated.^34^

In the doctrine, it is argued that, although other conditions are met, only the termination of pregnancy without the consent of a spouse is not subject to any sanction, and this situation is considered inappropriate on the grounds that it may violate the reproductive right of a spouse.^35^ However, in the case of the termination of pregnancy without consent, it is possible for a person to claim compensation from his pregnant spouse and other persons who participate in the termination of pregnancy.^36^ Therefore, it is not correct to say that there are no sanctions for the termination of pregnancy without the consent of a spouse.

#### II.A.3. Authorization Certificate

It has been stated in the PPL that a permission certificate must be obtained from those who accept the termination of pregnancy, and the principles of filling out this document in its form are indicated in the regulation to be issued (Art. 5, Para. 4). According to the Regulation, persons who are required to consent to the termination of pregnancy must sign a consent form when they apply for the procedure (Art. 15, Sentence 1).

The annex to the Regulation also contains a copy of the authorization (Art. 13, Para. 1). Accordingly, a physician must explain to persons who must consent to the procedure the medical consequences, possible complications, and the gravity and importance of the procedure. The physician must also explain that this procedure cannot be performed without consent and that the scope and subject matter of the consent will not be exceeded without medical necessity prior to signing the document by writing the date. Those who give consent must also sign and date the document, signify their understanding of the explanations made by a physician in detail, and indicate their acceptance of the procedure without any violence, threat, suggestion, or material and moral pressure. They must also indicate that they are aware of their responsibility and that they will not use the consequences of the procedure against each other and against the physician and the hospital.

According to the Regulation, in the absence of a spouse or guardian, it is sufficient to submit a signed document stating that they consent to the termination of pregnancy. In this case, the person who brought the document has to sign a separate document indicating that she accepts the legal responsibility of the document (Art. 15, Sentences 2 and 3). As there is a specific regulation here only for the absence of a spouse or guardian, it can be said that a pregnant person with the power of discernment and her guardian, if any, must sign the warrant document in the presence of a physician after being informed by the latter. Certainly, this cannot be imposed on a pregnant person who refuses to be informed.^37^ As a matter of fact, according to the Patient Rights Regulation (PRR), a patient may request not to be provided with information (PRR Art. 20^38^) or request that someone else be informed instead of themselves (PRR Art. 18, Para. 3^39^). However, even in cases in which a pregnant person refuses to be informed, it would be appropriate to provide her with at least the most basic information, such as the fact that her pregnancy cannot be terminated if she does not want it to be.^40^

The part in the sample of the consent document, which states that the people who are required to give consent must declare that they accept the termination of pregnancy without any violence, threat, suggestion, or material and moral pressure, and that they will not use the consequences of this procedure against each other, the physician, and the hospital, is also invalid.^41^ Of course, all the rights that these persons may claim are reserved in cases where they actually accept the termination under force or threat and in cases where a negative result arises because of malpractice. According to the TCC, no one can give up their rights and capacity to act, even partially. No one may also deprive others of their freedoms or restrict them in a manner contrary to law or morality (Art. 23, Paras 1 and 2).

According to the sample consent document, this document must be signed by parents serving as guardians or, in case of disagreement, by the father; if the father is dead or absent, this can be signed by the mother. This rule of the Regulation, which gives precedence to the father’s opinion over the mother’s, is contrary to the Constitution and the TCC. Indeed, according to the Constitution, the family, which is the foundation of Turkish society, is based on equality between spouses. According to the TCC, as long as marriage continues, parents must jointly exercise parental authority. In the event of the termination of a common life or separation, judges may award custody to one of the spouses. Custody belongs to the survivor in case of the death of one of the parents, and to a party to whom a child is left in case of a divorce (Art. 336). If the parents are not married, then the mother has custody. However, if a mother is a minor, restricted, or deceased, or if custody has been taken away from her, judges may appoint a guardian or grant custody to a father according to the best interests of a child (Art. 337). In the face of these provisions of the Constitution and the TCC, the Regulation, which is hierarchically inferior to them, has no applicability.^42^ Therefore, in cases where both parents have custody, they must both consent to the termination of their child’s pregnancy. However, in cases where only one of the parents has custody, the pregnancy may be terminated only with the consent of that parent.

According to the example of the permission document, illiterate persons can simply press their left-hand thumb on a document instead of signing it. In fact, issues related to signs that substitute for signatures are not the subject of this Regulation. Presumably, such a rule is included in the Regulation to guide practitioners. Furthermore, it is also understood from the sample of the authorization document that if a pregnant person is under guardianship, a certified copy of the decision issued by the magistrate must be attached to the authorization document.

#### II.A.4. Person Who Can Perform the Termination of Pregnancy and the Place Where the Procedure Will Be Performed

Termination of pregnancy can only be performed by a physician. Indeed, according to the Regulation, uterine evacuation can only be performed by gynecologists and obstetricians. However, general practitioners who have taken courses in training centers opened by the Ministry of Health and obtained a qualification certificate may also perform uterine evacuation by menstrual regulation method under the supervision of a gynecologist and obstetrician (Art. 3, Paras 2 and 3). As it is difficult to find such medical practitioners in some rural areas, it is not appropriate to require general practitioners with a certificate of competence to perform procedures under their supervision.^43^

Termination of pregnancy is performed by gynecologists and obstetricians in places where they practice their profession and by general practitioners in official treatment institutions. Uterine evacuations requiring anesthesia are performed in public and private hospitals where anesthesia can be administered (Regulation Art. 4, Paras 1 and 2). Furthermore, it is mandatory to have the tools and equipment enumerated in List No. 1^44^ attached to the Regulation in the official treatment institutions and private hospitals where the termination of pregnancy will be carried out, as well as in the clinics of gynecologists and obstetricians (Regulation Art. 4, Para. 3).

Since 2005, the Model List of Essential Medicines published by the WHO^45^ has included a medicine inhibiting the secretion of the hormone progesterone, which maintains pregnancy, and a medicine that increases contractions in the uterus and helps remove the residues caused by uterine contractions.^46^ However, such a list unusually includes the phrase ‘where permitted by national law and where culturally acceptable’ right next to these medicines.^47^ In Turkey, it is not possible to prescribe these medicines for the voluntary termination of pregnancy.^48^ Although voluntary termination is allowed, it is not appropriate that these drugs, which are found to be extremely safe in studies^49^ and terminate pregnancy in much simpler ways than other methods,^50^ cannot be used. However, these drugs can be accessed illegally^51^ and can cause various health problems for pregnant people and embryos/fetuses when they are used unconsciously without consulting a physician.^52^

### II.B. Termination of Pregnancy Because of an Anomaly or a Disease (PPL Art. 5, Para. 2)

According to the PPL, if the gestation period is more than 10 weeks, the uterus may be evacuated only if the pregnancy poses or will pose an imminent threat to the mother’s life or cause severe disability for the child to be born and the generations that follow, with a reasoned report based on objective findings by a specialist in obstetrics and gynecology and a specialist from the relevant field (Art. 5, Para. 2).

According to the provision, it is the duty and authority of physicians to determine cases where pregnancy threatens or will threaten the life of a pregnant person or will cause severe disability for a child to be born and the generations to follow. However, in List No. 2^53^ attached to the Regulation, such anomalies and diseases are listed one by one. Although a fairly large enumeration has been made here, various anomalies and diseases that may be found in the PPL Art. 5, Para. 2 are not yet included in this list. Since 1983, when the Regulation first came into force, the diagnostic possibilities have improved significantly, and more and more anomalies or diseases can be diagnosed in the prenatal period.^54^ In addition, in cases where a pregnant person suffers from depression because of pregnancy, which is not listed here, it is not possible to say that her life is not and will not be in danger because of the continuation of the pregnancy. As a matter of fact, the rate of attempted suicides in these cases is quite high.^55^

In any case, the purpose of such an enumeration is to provide an assurance to physicians and to eliminate their hesitations regarding the termination of a pregnancy that exceeds 10 weeks.^56^ However, if a disease or anomaly not included in this list also threatens or will threaten the life of a pregnant person or will cause severe disability for a child to be born and generations to follow, it is not unlawful to terminate the pregnancy upon the fulfillment of other conditions.^57^ At the same time, refraining from terminating a pregnancy when it should be terminated, simply because the anomaly or disease is not included in this list, makes the physician responsible for the negative consequences of the continuation of the pregnancy. As mentioned previously, the law is hierarchically above the charter, and the unlawful by-law provision is not applied. However, it is unrealistic to expect all physicians to know this principle and act accordingly.

Although it is argued in the doctrine that this list in the Regulation should be updated and sample lists created, considering the rapid developments in the field of medicine, it is understood that the updated list will also lose its meaning in a short period of time.^58^ Therefore, contrary to what is desired, it would be appropriate to completely remove this list from the Regulation, which makes physicians’ situations quite uncertain.^59^ In English law, the authority to determine in good faith whether there are anomalies or diseases that would justify the termination of pregnancy belongs to two physicians (United Kingdom Abortion Act 1967 Art. 1, Para. 1), and no attempt has been made to specify what these are.^60^

As there is no time limit for termination of pregnancy based on the reasons set out in the PPL Art. 5, Para. 2, it is possible to evacuate the uterus until the pregnancy is terminated. The reason why no time limit is included here is that some anomalies and diseases occur during late pregnancy periods.^61^

In the doctrine, although compatible with life, there is an opinion that a pregnancy should not be terminated after the viability limit is exceeded in the presence of diseases or anomalies that will cause severe disability for an embryo/fetus and generations to follow.^62^ In actual practice, although compatible with life, it is, in fact, observed that some physicians refrain from terminating pregnancies in the presence of diseases or anomalies that will cause severe disability for an embryo/fetus and generations to follow, either completely or after the viability limit has been exceeded.^63^ However, the Law clearly makes a distinction here not according to whether or not the anomaly or disease is compatible with life, but according to whether it causes a severe disability; it also does not include any limit in terms of duration. In addition, there are very strong arguments stating that viability, the boundaries of which cannot be clearly defined,^64^ cannot constitute a legitimate basis for regulations on the termination of pregnancy.^65^

To terminate pregnancy based on the reasons specified in the PPL Art. 5, Para. 2, the permission of the persons who have to consent to the termination of a pregnancy not exceeding 10 weeks must be obtained, except the spouse.^66^ As a matter of fact, although a regulation is made in the PPL Art. 6, Para. 1^67^ regarding the leave to terminate pregnancy, including termination because of anomalies or diseases and all the interventions specified in the PLL Art. 5, it is stipulated that the consent of the pregnant person’s spouse will be sought only in the voluntary termination of pregnancy (PLL Art. 5, Para. 1) in the PLL Art. 6, Para. 2^68^. However, it would be more accurate to accept that pregnancy can be terminated only with the consent of a pregnant person, without the need for the consent of the legal representative and the magistrate, in cases involving minors or limited persons who have the power to distinguish. As will be explained in detail below, in emergency situations where the life of a pregnant person is in danger, it should be accepted that it is not necessary to obtain consent from anyone, including the pregnant person herself.

Pregnancy can be terminated for the reasons specified in the PPL Art. 5, Para. 2, but only with the reasoned reports of an obstetrician and gynecologist, as well as a specialist from the relevant branch, based on objective findings. According to the Regulation, this report must be based on precise clinical and laboratory findings (Art. 5, Para. 2). The physician who terminates a pregnancy must send this report, together with the results of the procedure, to the health and social welfare directorates in the provinces and the government medical offices in the districts within 1 week after the procedure at the latest. These reports are collected at the provincial health and social assistance directorate (Art. 5, Para. 3).

According to the Regulation, uterine evacuation in pregnancies exceeding 10 weeks must be carried out in public inpatient treatment institutions and private hospitals (Art. 6, Para. 1). It is obligatory to have the tools, equipment, and personnel included in List No. 3^69^ attached to the Regulation (Art. 6, Para. 2).

### II.C. Termination of Pregnancy in Emergency Situations (PPL Art. 5, Para. 3)

According to the PPL, in emergency situations that threaten life or one of the vital organs if not intervened immediately, then the uterus shall be evacuated by an authorized physician who detects the situation (Art. 5, Para. 3, Sentence 1). The circumstances requiring urgent intervention are not listed in the PPL, and their specification is left to the Regulation (PPL Art. 5, Para. 4).

According to the Regulation, even if the cervical internal os is closed, the uterus should be evacuated urgently in cases where there is vaginal bleeding that may endanger the life of the pregnant person, when the cervical internal os is open, a part of the pregnancy product in the uterus falls and the bleeding continues, or when there is a risk of infection (Art. 8). In our opinion, it is not appropriate to make such an enumeration, which may cause physicians to hesitate in other emergencies that threaten life or one of the vital organs. In such a situation, however, the best person to make such a determination is the attending physician, who should not refrain from terminating the pregnancy, if necessary. In the PPL, there is no time limit for the termination of pregnancy because of emergencies; thus, it is possible to evacuate a uterus until the pregnancy is terminated.

A regulation has been made regarding all interventions specified in Article 5, including emergency situations, in the PPL Art. 6, Para. 1 regarding permission to initiate termination of pregnancy: the consent of a pregnant person, her guardian, or her guardian and the magistrate is required to terminate a pregnancy, depending on the situation. Only in the PPL Art. 6, Para. 3 is it stated that permission is not required in certain cases: when parents’ permission is sought, when the magistrate requires time, and when the case involves an emergency that threatens life or one of the vital organs unless promptly intervened. These regulations create the impression that it is not possible to terminate a pregnancy without the consent of a pregnant person who has the power of discernment, the legal representative, or the magistrate, even in the presence of urgent circumstances, and that a pregnancy can be terminated without their consent only in cases where it requires time to obtain immediate permission from parents or guardians (although not explicitly stated in the provision) and the magistrate.^70^

Certainly, the condition that renders lawful the medical intervention to the patient who has full capacity (ie has the power of discernment and is an adult but not restricted) is the consent given by the patient after being informed by a physician. However, if not terminating the pregnancy immediately leads to the death of a patient in this situation, it is against the law to allow her to die by not terminating her pregnancy upon her request. In fact, according to the TCC, no one can give up their rights and capacity to act, even partially. No one may renounce her freedoms or restrict them contrary to law or morality (Art. 23, Paras 1 and 2). According to the PRR, euthanasia is prohibited; the right to life cannot be renounced on the grounds of medical necessity or by any means whatsoever. No one’s life can be terminated, even at their own or another person’s request (Art. 13). Termination of pregnancy in emergency cases serves the best interests of a patient and also means the exercise of the authority arising from the exercise of the medical profession.^71^ According to the TCC Art. 24, Para. 2, in addition to the consent of a person whose personal right is violated, the use of superior private benefit and authority given by the law makes the intervention with personal rights legal. Pursuant to the RMD, a physician, regardless of their position and specialization, shall render first aid in emergency cases where the necessary care cannot be provided, unless there is a force majeure (Art. 3, Para. 1).^72^ According to the TPC, no penalty is imposed on a person who exercises their right (Art. 26, Para. 1). Within the framework of all these regulations, it should be accepted that the physician who terminates the pregnancy against the consent of the patient in the presence of emergencies has neither legal nor criminal liability.^73^

In cases where consent cannot be obtained from a fully competent patient because of unconsciousness, the element that makes the medical intervention performed to save the patient’s life in compliance with the law is both the patient’s best interest and the exercise of the authority arising from the exercise of the medical profession. In a situation in which the pregnancy can be terminated even against the consent of a patient who is already conscious, it should not be possible for the patient’s unconsciousness to create a different result.

Before a pregnancy is terminated, the consent of those who have the power of discernment but who are not yet adults or who have limited capacity is also obtained. However, as mentioned previously, the PPL inappropriately requires the consent of legal representatives of pregnant people in this situation, and even the magistrate for those under guardianship, before the termination of pregnancy. Fully incapacitated persons who are permanently deprived of the power of discernment are not required to consent to termination, which makes no sense anyway. For persons in this situation, those who must consent to the termination of pregnancy are the parents or guardians of a pregnant person and the magistrate, as the case may be. However, in all these cases, what makes the procedure lawful is not the permission of the legal representatives and judges but the fact that the medical intervention serves a patient’s best interest and is an exercise of the authority arising from the practice of the medical profession. The authorization by legal representatives and judges only creates a presumption that the intervention serves the best interests of a patient.^74^

As a result, in cases where it is urgently necessary to terminate pregnancy to save the life of a pregnant person, it should not be necessary to obtain permission from anyone, including the pregnant person, within the framework of the prohibition of euthanasia, the patient’s best interests, and the exercise of the right arising from the practice of the medical profession. In such cases, the life of a pregnant person may be given precedence over the life of an embryo/fetus in termination procedures. Indeed, the embryo/fetus may not yet be viable at the time of termination. However, it would not be legally wrong to accept that the life of a pregnant person who already has personality is legally superior to that of an embryo/fetus, which does not yet have it.^75^

Furthermore, physicians are obliged to notify the health and social welfare directorates in provinces and government medical offices in districts of the identity of a pregnant person, the intervention performed, and the reasons necessitating the intervention before terminating a pregnancy in urgent cases or, if this is not possible, within 24 h at the latest after the intervention (PPL Art. 5, Para. 3, Sentence 2). According to the Regulation, these reports are collected at provincial health and social assistance directorates (Art. 9, Para. 2).

In cases of emergency, as mentioned previously, uterine evacuation is performed by gynecologists and obstetricians (Regulation Art. 7, Para. 2). This intervention must be carried out in public inpatient facilities and private hospitals. However, if it is not possible to move a pregnant person to these places, this intervention can also be performed in places where the patient is present, such as a doctor’s office or home (Regulation Art. 9, Para. 1).

### II.D. Termination of Pregnancy Because of Conception as a Result of a Crime of which the Pregnant Person Is the Victim (TPC Art. 99, Para. 6)

Crimes related to the termination of pregnancy in the TPC, including illegal abortion (Art. 99) and abortion (Art. 100), are regulated in detail under the headings. However, because a child is defined in the TPC as a person who has not attained the age of 18 (Art. 6, Sub-Para. b), the use of the term ‘child’ is not appropriate here. It is clear that the term ‘child’ in these provisions should be understood as an embryo/fetus.^76^

According to the TPC, if a woman with a gestational age of more than 10 weeks voluntarily aborts her embryo/fetus, she is sentenced to imprisonment for up to 1 year or imposed a judicial fine (Art. 100). Anyone who aborts a woman’s embryo/fetus without her consent shall be punished by imprisonment for 5–10 years (Art. 99, Para. 1). If this act causes the woman to suffer physical or mental health damage, the person is punished with imprisonment from 6 to 12 years; if the act causes the death of the woman, the person is punished with imprisonment from 15 to 20 years (Art. 99, Para. 3). Even if there is no medical necessity, or even if it is based on consent, a person who aborts the embryo/fetus of a woman whose gestation period is more than 10 weeks is sentenced to imprisonment from 2 to 4 years. If this act causes the woman to suffer physical or mental health damage, the person is punished with imprisonment from 3 to 6 years; if the act causes the death of the woman, the sentence entails imprisonment for 4–8 years (Art. 99, Para. 4). In this case, the woman who consents to abortion is also sentenced to imprisonment of up to 1 year or a judicial fine (Art. 99, Para. 2–Art. 100). Even if it is based on consent, if the embryo/fetus of a woman whose gestation period has not completed 10 weeks is aborted by an unauthorized person, she is sentenced to imprisonment from 2 to 4 years. If the acts defined in the first four paragraphs of the TPC Art. 99 are committed by an unauthorized person, the penalty to be imposed according to these paragraphs is increased by half (Art. 99, Para. 5).

According to the sixth and last paragraph of the TPC Art. 99, if a woman becomes pregnant as a result of a crime of which she is the victim, no penalty shall be imposed on the person who terminates the pregnancy, provided that the gestation period is not more than 20 weeks and the woman’s consent is obtained. However, in this situation, the pregnancy must be terminated in a hospital setting by specialized physicians.

Under the TPC Art. 99, Para. 6, some of the crimes that a pregnant person may be a victim of are the qualified versions of sexual assault (TPC Art. 102) and sexual abuse of children (TPC Art. 103), as well as sexual intercourse with a minor (TPC Art. 104). One of the issues that should be emphasized here is that the perpetrator of the crime of sexual assault, which is committed by inserting an organ or other object into the body in a way that may lead to pregnancy, may also be the spouse of the victim. In this case, the investigation and prosecution are dependent on the complaint of the victim (TPC Art. 102, Para. 2, Sentence 2). However, the lack of investigation or prosecution because of the victim’s failure to file a complaint against her spouse or the withdrawal of her complaint later does not change the fact that this is considered a criminal act. Therefore, the pregnancy can be terminated, even if the victim has not filed a complaint against her spouse or has later withdrawn the complaint. In other words, the wish to terminate the pregnancy and the wish not to punish the spouse are independent of each other.

Except for crimes against sexual inviolability, it is possible to terminate pregnancy within the scope of the TPC Art. 99, Para. 6 in cases where pregnancy is because of crimes committed during artificial insemination. For example, if a healthcare worker intentionally or negligently performs artificial insemination using sperm cells belonging to someone else and not those taken from the patient’s spouse, this person commits the crime of misconduct as stipulated in the TPC Art. 257, and the pregnancy resulting from such crime can be terminated under the TPC Art. 99, Para. 6.^77^

Although it is forbidden in Turkey for relatives of a certain proximity to marry each other^78^ and sexual intercourse between such persons is considered immoral, such (incestuous) relationships consensually entered into by consenting adults do not constitute a crime. Therefore, it is not possible to terminate a pregnancy resulting from such a relationship under the TPC Art. 99, Para. 6.^79^ In some legal systems, the occurrence of pregnancy as a result of such a relationship is accepted as a situation in which pregnancy can be terminated in itself.^80^

According to the TPC Art. 99, Para. 6, another condition that must be met so that the termination of pregnancy is not punished is that the gestation period must not exceed 20 weeks. In other legal systems that recognize the right to terminate pregnancies resulting from criminal acts, periods longer than 20 weeks (usually 22 weeks) are adopted.^81^

In cases where the termination of a pregnancy exceeding 10 weeks is requested on the grounds that the pregnancy is caused by a criminal offense, it is clear that the physician may hesitate as to whether the pregnancy may be terminated. In fact, there is no clear regulation in the TPC regarding the conception of a woman as a result of a crime and who will decide to terminate this pregnancy. Of course, it is the duty of the courts to detect the existence of a crime. However, as the stages of the proceedings (investigation and prosecution) can take a long time, seeking a finalized court judgment establishing the existence of the crime in this case is not required under the TPC Art. 99, Para. 6 and may render its application impossible. There is a 20-week time limit to terminate the pregnancy, and within this period, a crime is usually still at the investigation stage.^82^

In practice, during the investigation phase, it is generally requested that the person claiming to be pregnant as a result of a crime be examined and a forensic medicine report drawn up with the decision of the magistrate, and in cases where delay is inconvenient, the public prosecutor (Code of Criminal Procedure Art. 76^83^—PRR Art. 22, Paras 2–3^84^), the magistrate decides whether the pregnancy can be terminated by taking this report into account.

In German law, it is accepted that the physician should directly decide whether a pregnancy can be terminated (German Criminal Code Art. 218a, Para. 3). To initiate termination, it is sufficient for the physician to consider it highly probable that the pregnancy occurred as a result of a crime within the framework of their medical knowledge.^85^ Although it is argued that it would be appropriate to include a similar regulation in Turkish law,^86^ it seems more appropriate to leave this decision to the judges, because they can access other evidence such as witness statements in addition to the medical evidence that is also available to physicians. In fact, even if a short time has passed after the crime has been committed, the possibility of obtaining medical evidence showing that the person has been exposed to the crime may have already disappeared.^87^

Setting aside the question of how the TPC Art. 99, Para. 6 should be amended, what a physician who is requested to terminate a pregnancy has to do is to contact the judicial authorities conducting the investigation. In addition, if their examination reveals findings indicating exposure to the crime, they must also report them. If the incident has not already been reported to the judicial authorities, then they are required to first report it to the judicial authorities. Otherwise, they commit the crime of failing to report a crime. As a matter of fact, according to the TPC Art. 280, while performing their duty, healthcare professionals (physicians, dentists, pharmacists, midwives, nurses, and other persons providing healthcare services) who fail to notify the competent authorities or delay in doing so despite having an indication that a crime has been committed shall be sentenced to imprisonment for up to 1 year.

In Turkey, even judicial authorities may hesitate when faced with a request for the termination of a pregnancy resulting from a criminal offense and try to pass the buck to another authority by refraining from making a decision. In a decision rendered as a result of an individual application, the Constitutional Court has ruled that the failure to decide on the request for termination of pregnancy within a reasonable period of time by dragging out the request is a violation of the right to protect and develop the material and moral existence regulated in the Constitution Art. 17, Para. 1, and has ordered nonpecuniary damages in favor of the person who had to give birth to a child by missing the 20-week period because of the hesitation of the judicial authorities.^88^

A significant part of the judicial authorities’ hesitation in this regard stems from the lack of clarity in the TPC. On the one hand, although the title of the TPC Art. 104 is ‘Sexual intercourse with a minor’, the first paragraph of this provision stipulates that anyone who has sexual intercourse with a child who has completed the age of 15, without force, threat, or deception shall, upon complaint, be sentenced to imprisonment from 2 to 5 years. It is debated whether this crime can be committed against a child who is made an adult by marriage^89^ or court decision^90^ because of the title of the provision.^91^ The Turkish Court of Appeals (Yargıtay), on the other hand, have developed a jurisprudence in which this crime cannot be committed against a child who has become an adult.^92^ Again, if both people who have consensual sexual intercourse have completed the age of 15 but are not yet adults, if it is accepted that the crime occurred, which party will be considered the perpetrator is also controversial in the doctrine.^93^ The Court of Appeal accepts that a crime has occurred in such cases, but with patriarchal thought patterns: it has developed a jurisprudence that the man is the perpetrator and the woman is the victim,^94^ and in a relationship between two men, the active party is the perpetrator, and the passive party is the victim.^95^

If all these discussions are put aside and considered in the context of the right to terminate a pregnancy, it would be appropriate to accept the fact that the pregnant person is a child in a situation in which pregnancy can be terminated, thus eliminating all hesitations in this regard. The same conclusion can be reached if we consider the problems that can arise from childbearing, particularly the high likelihood of complications in such a pregnancy.^96^ In Finnish law, when a pregnant person is under the age of 17, this is recognized as a situation in which the pregnancy can be terminated in itself. In fact, it is even accepted here that the pregnancy can be terminated in cases where the pregnant person is over 40 years of age (Law on The Interruption of Pregnancy Art. 1, Para. 4).

According to the TPC Art. 99, Para. 6, the consent of the pregnant person is necessary and sufficient for the termination of pregnancies resulting from a crime. Given that the continuation or termination of a pregnancy resulting from a crime takes place on the pregnant person’s body and therefore affects her the most,^97^ because the perpetrator of the crime that resulted in pregnancy may be the pregnant person’s spouse, and the pregnant person may want to hide the situation even from her closest loved ones for fear of violence, especially stigmatization, this regulation, which only requires the consent of the pregnant person, is considered appropriate. Of course, the pregnant person must have the power of discernment to consent to the termination of her pregnancy. However, in cases where the pregnant person does not have the power of discernment (ie she is fully incapacitated), the person whose consent will be sought in this regard is the parent or guardian of the pregnant person and the magistrate.^98^

## III. THE PROBLEM OF INACCESSIBILITY OF THE RIGHT TO TERMINATE PREGNANCY

According to the 2020 Report on Abortion Services in Public Hospitals in Turkey (hereafter referred to as ‘Report’) published by the Kadir Has University Gender and Women’s Studies Research Center,^99^ it is understood that many public hospitals refrain from providing services for termination of pregnancy (especially voluntary termination of pregnancy) under various pretexts, such as the unavailability of equipment and personnel, as stipulated in the Regulation. This Report, together with the Center’s 2016 Report on the same topic,^100^ shows that public hospitals have adopted the COVID-19 pandemic as a new excuse to avoid termination of pregnancy. However, it is clear that this is a basic health service that should be provided, even during pandemics.^101^

This attitude of public hospitals significantly hinders the exercise of the right to termination of pregnancy. According to the Social Security Institution Health Implementation Communiqué, pregnant people who cannot receive this service from public hospitals within the scope of social security must apply to private hospitals or medical practices. A large number of pregnant people who do not have the means to do so try to terminate their pregnancies under cheap but medically inappropriate conditions, which may lead them to lose their health and even their lives.^102^ A 2016 report on Turkey by the United Nations Committee on the Elimination of Discrimination against Women also drew attention to this issue and recommended that the State strictly inspect public hospitals.^103^ It is essential that this recommendation be followed as soon as possible to prevent discrimination between rich and poor and to prevent pregnancy-related deaths and injuries.

Although this unlawful situation, which occurs spontaneously in practice, has not yet been resolved, it is argued that in Turkish law^104^—as in some legal systems—it is necessary to grant healthcare professionals the right to abstain from participating in procedures for the termination of pregnancy for conscientious reasons.^105^ It is, of course, debatable whether it is appropriate to grant healthcare workers the right to refrain from participating in such procedures for reasons of conscience. However, for this right to be recognized and, more importantly, to eliminate the unlawful situation that arises spontaneously in practice, it must first be ensured that every pregnant person can receive this service from a health institution that she can easily access within the scope of social security. This point was also emphasized by the ECtHR in a judgment on conscientious objection.^106^

Clearly, allowing the prescription of safe drugs to terminate a pregnancy, as mentioned earlier, would also make a significant contribution to making the right to terminate a pregnancy accessible. During the COVID-19 pandemic, instead of allowing this to be used as an excuse to avoid providing termination of pregnancy services, allowing these drugs to be administered by the pregnant person herself at her place of residence after a consultation between her and her physician by means of remote communication, as is the case under English law (Abortion Act 1967 Art. 1, Para. 3/D)^107^ would be accurate. Although the opportunity for a good start has been missed, there is still an opportunity to do what is necessary from now on.

## IV. CONCLUSION

To be able to speak of the full recognition of the right to terminate pregnancy in a legal system, certain minimum conditions must be met. Accepting that a pregnancy can be terminated voluntarily is among the most important of these conditions. The period during which this right can be exercised should not be kept short in a way that jeopardizes the essence of the right. In Turkish law, the period during which the right can be exercised is determined as 10 weeks. However, it is clear that this period is not suitable for the effective use of the right. For this reason, it would be appropriate to extend the 10-week period to at least 12 weeks.

In addition, because the continuation of the pregnancy and the birth of the child against the consent of the pregnant woman are not acceptable, it should be accepted that only the will of the pregnant woman who has the power to distinguish is sufficient for the pregnancy to be terminated. In addition to the pregnant woman, the consent of her spouse, legal representative, or official institutions and organizations to which she is subject should not be sought. For this reason, the regulations in Turkish law that seek the consent of persons other than the pregnant woman, who have the power to discriminate to terminate the pregnancy, should be abolished first.

In an ideal system, certain obligations should be avoided, such as receiving counseling service that can be used as a pressure tool before the pregnancy is terminated and then waiting for a certain period of time to increase the costs of termination of pregnancy. It is correct that such obligations are not included in Turkish law, and proposals for the introduction of such obligations should not be taken into account in the future.

Furthermore, in an ideal system, it is also very important not to make regulations that would cause practitioners to hesitate. Some regulations in Turkish law are unsuccessful in this respect. In particular, although it is stated in the Law that pregnancy can be terminated in cases where the pregnancy threatens or will threaten the mother’s life or causes severe disability for the child to be born and the generations that follow it, these anomalies and diseases are listed one by one in the Regulation. However, this census, which has lost its meaning as new anomalies and diseases become diagnosable over time, can make physicians hesitate. In addition, although it is accepted in Turkish law that pregnancies resulting from crimes can be terminated, there are hesitations in practice, as the law does not clearly state who will make this decision. As a result, these hesitations may lead to situations in which the right to terminate pregnancy can no longer be exercised by missing the period stipulated in the Law, resulting in the birth of an ‘undesirable child’.

Although the State is obliged to organize services for the termination of pregnancy (a basic health service) in a way that is easily accessible to everyone who needs it, Turkey is not very successful in this regard. Thus, allowing the use of drugs that safely and easily abort pregnancy will greatly contribute to the State’s fulfillment of this obligation.

## Footnotes

1 To be able to speak of the occurrence of full birth, a child’s body must be completely separated from the pregnant person. Andreas Kley and Goran Seferovıc, in Orell Fusslı Kommentar: Zgb Kommentar Schweızerısches Zıvılgesetzbuch 150 (Jolanta Kren Kostkiewicz et al. eds., 2016). It does not matter whether this separation occurs naturally or by an outside intervention. Heınz Hausheer and Regına E. Aebı-Muller, Das Personenrecht Des Schweızerıschen Zıvılgesetzbuches 15 (2008). It is also not necessary that the umbilical cord is cut. Jurgen Ellenberger, in Beck’Iche Kurz Kommentare Band 7: Palandt Burgerlıches Gesetzbuch 11 (2014).

2 To be able to speak of the occurrence of a right birth, a child must live independently of the pregnant person, even for a very short moment. For the detection of this condition, it is enough to see any signs of life in the child. Klaus Saerbeck, Begınn Und Ende Des Lebens Als Rechtsbegrıffe 36 (1974).

3 To acquire a personality, it is not necessary to have a condition other than being born full and alive, especially for a child to have the ability to live. Peter Tuor, Bernhard Schnyyder, Jorg Schmıd and Alexandra Rumo-Jungo, Das Schweızerısche Zıvılgesetzbuch Begrundet Von Peter Tuor 125 (2010). In addition, it is not their appearance that gives a human being the quality of a human being, but the fact that a human being gave birth to them. Therefore, it does not matter if the view of nature deviates from that of any human being from a standard human being. Cem Baygın and Ahmet Nar, Medenİ Hukuk Derslerİ - I: BaşlangıçHükümlerı ve Kİşİler Hukuku 307 (2022).

4 Dilşad Keskin, *Doğum Öncesi Gerçekleşen Zarar Verici Fiil Sebebiyle Tazminat: Ceninin Tazminat Talebi*, 17 Gazı University Faculty of Law Review 743, 746 (2013). Compare: Bİlge Öztan, Kİşİler Hukuku: Gerçek Kİşİler 52 (2021).

5 Accepting that the capacity to have rights begins not at the moment of full and rightful birth, but before it, allows a person to acquire rights arising from law on successions. See: Leyla Müjde Kurt, *Ceninin Malvarlığı Hakları*, 2 Inonu Unıversıty Law Revıew 177, 185 et seq. (2011). To fully protect the inheritance rights of persons born through artificial insemination, it is necessary to accept that these persons acquire their legal capacity not at the moment of conception, but at the moment at which the medical process is initiated. Also see: Bahar Öcal Apaydın and Nurten İnce Akman, *Dondurularak Saklanmış Üreme Hücresinin veya Embriyonun Eşin Ölümünden Sonra Ana Rahmine Transferi (Postmortal Döllenme) Sonucunda Doğan Çocuğun Mirasçılığı*, 24 Ankara Hacı Bayram Veli Unıversıty Faculty of Law Revıew 63, 92 et seq. (2020). Compare: Yusuf Büyükay, İstenmeden DünyayaGelen Çocukların Bakım Eğıtım Ve Tedavİ Gİderlerİnİn Tazmİn Edılmesİ Sorunu 50 (2006).

6
Rona Serozan, Medenİ Hukuk: Genel Bölüm/Kİşİler Hukuku 424 (2015).

7 The liberal approach explains that the woman can freely dispose of an embryo/fetus, which is considered part of her body, on the grounds that it is a private privilege regarding her private life. Also see: Catharıne A. Mackınnon, Toward A Femınıst Theory of the State 184 (1991).

8 Türkiz Gökgöl, *İsteyerek düşük olgusuna global bakış: Yasalar ve Deneyimler*, 10 Turkısh Journal of Publıc Health 1, 4–5 (2012).

9 See: Center for Reproductive Rights, *The World’s Abortion Laws*, https://reproductiverights.org/maps/worlds-abortion-laws/ (accessed Jul. 12, 2022). For example: Switzerland Criminal Code Art. 119, Para. 2.

10 In a similar opinion: Candan Yılmaz, *Gebeliğin Sonlandırılmasında İzin Verilen Yasal Sürelere İlişkin Karşılaştırmalı İnceleme*, 21 Dokuz Eylul University Faculty of Law Review 3311, 3322 (2019).

11 Also see: Ronald Dworkin, Life’s Dominion: An Argument about Abortion, Euthanasia, and Individual Freedom 35 et seq. (1993).

12
Presidency of Religious Affairs, Türkİye’de Dınî Hayat Araştirmasi, 3 et seq. (2014) (accessed Sept. 18, 2022).

13 High Board of Religious Affairs, *Kürtaj yaptırmak caiz midir?* (accessed Sept. 19, 2022).

14 For detailed information on this and other fatwas on the subject in the Hanafi Sect, see: İbrahim Tüfekçi, *İslam Hukukuna Göre Gebeliğin Sonlandırılması*, 45 International Journal of Theological And Islamic Studies 111, 135 et seq. (2013).

15 For example, German Criminal Code Art. 218a, Para. 1, Sub-Para. 1.

16 Şule Özel, Yaprak Engin Üstün and Filiz Avşar, *Türkiye’de Gebelik Terminasyonunun Yasal Durumu*, 14 the Journal of Gynecology-Obstetrics and Neonatology 34, 35–6 (2017).

17 Özlem Özkan, *Ülkelere Göre Kürtaja Yönelik Yasal Düzenlemelere Genel Bir Bakış*, 27 Community and Physician 304, 305 (2012).

18 See: Guttmacher Institute, *Counseling and Waiting Periods for Abortion* (accessed Nov. 18, 2022).

19 Terry Nicole Steinberg, *Abortion Counseling: To Benefit Maternal Health*, 15 American Journal of Law & Medicine 483, 495–6 (1989).

20 Deborah Karasek, Sarah C. M. Roberts and Tracy A. Weitz, *Abortion Patients’ Experience and Perceptions of Waiting Periods: Survey Evidence before Arizona’s Two-visit 24-hour Mandatory Waiting Period Law*, 26 Women’s Health Issues 60 (2016).

21
Mustafa Dural and Tufan Öğüz, Türk Özel Hukuku Vol. 2: Kİşİler Hukuku 121 fn. 350 (2021).

22 Also see: Rosa Hinzpeter-Schmidt, *Die Einwilligung einer Minderjährigen in den Schwangerschaftsabbruch*, Juristische Arbeitsblätter 705, 711 (2022).

23 See: https://www.who.int/publications/i/item/9789240039483 (accessed Oct. 11, 2022), 42 et seq.

24 Compare: Recep Doğan, *Women’s Reproductive Rights, Abortion, The Crime of Self-Induced Abortion and Causing Miscarriage*, 29 Union of Turkish Bar Associations Review 73, 104 et seq. (2016).

25 Also see: Law on the Practice of Medicine and Medical Arts (LPMMA) Art. 70, Para. 1, Sentences 1 and 3: ‘Physicians, dentists and dentists get the consent of their parents or guardians in the first place for any kind of operation they will do. …(Consent is not required if he/she does not have or does not have a parent or guardian.)’; Patient Rights Regulation (PRR) Art. 24, Para. 1, Sentences 2 and 3: ‘…If the patient is small or confined, permission is taken from his/her parent or guardian. In cases where the patient is not present or has no parent or guardian, this condition is not sought’.

26 For other legal systems, see: Center for Reproductive Rights, *supra* note 9.

27 Helin Rojda İlaldı, *Kadının Menfaati Bakımından Rahim Tahliyesi,* 2 Maltepe University Faculty of Law Review 671, 688–9 (2020).

28 According to the Tcc, paternity is established between the child and the mother at birth (Art. 282, Para. 1). One of the ways by which the lineage between the child and the father is established is through the latter’s marriage with the mother (Art. 282, Para. 2). The father of a child born while a marriage is continuing or within 300 days of the end of the marriage is the spouse or the former spouse (Art. 285, Para. 1). The attachment of a child born after this period to the spouse or former spouse is possible with the proof that the mother became pregnant during the marriage (Art. 285, Para. 2).

29
*R.H. v Norway* (App. 17004/90).

30 Tansu Sayar and Yasemin Kılıç Öztürk, *Türkiye’de Uygulanan Kürtajda Eşin Rızasını Alma Zorunluluğu ve Amerikan Yargısından Bir Örnek: Planned Parenthood of Southeastern Pennsylvania v. Casey Kararı*, 20, Dokuz Eylul University Faculty of Law Review 321, 344 et seq. (2018).

31
*Supra* note 23.

32
*Concluding observations on the seventh periodic report of Turkey*, https://digitallibrary.un.org/record/840825 (accessed Sept. 10, 2022), 5.

33 Compare: Dogan, *supra* note 24, at 102 et seq.

34 Compare: Hüseyin Ertuğrul, *Türk Ceza Kanununda Çocuk Düşürme Suçu*, 6 Kirikkale University Journal of Social Sciences 361, 369 (2016).

35
*Id*. at 369–70.

36 Emel Badur, *Eşin Rızası*, 26 Union of Turkish Bar Associations Review 251, 298 (2013).

37 Sonia M. Suter, *The Politics of Information: Informed Consent in Abortion and End-of-Life Decision Making*, 39 American Journal of Law & Medicine 7, 55 (2013).

38 ‘Except where required by the provisions of the relevant legislation and/or measures to be taken by the competent authorities; a person may request that their relatives or no one be informed about his health status. In this case, that person’s decision is taken in writing. A patient can change their request for withholding information at any time and request that information be provided’.

39 ‘It is essential to inform the patient himself/herself. In the event that the patient requests that someone else be informed instead of him/herself, only the persons requested to be informed shall be informed, provided that this request is recorded in writing with the signature of the person’.

40 Also see: Regina Aebi-Muller, in Handkommentar Zum Schweizer Privatrecht: Personen und Familienrecht - Partnerschaftsgesetz Art. 1-456 Zgb—Partg 107 (Peter Breitschmid and Alexandra Jungo eds., 2016).

41 Also see: Alper Uyumaz and Yasemin Avcı, *Türk Hukuku'nda Gebeliğin Sonlandırılması*, 7 Inonu Unıversıty Law Revıew 579, 617–8 (2016).

42 Turhan Feyzioğlu, *Kanuna Aykırı Tüzükler Karşısında Mahkemelerin Durumu*, 6 Ankara Üniversitesi Hukuk Fakültesi Dergisi 27, 42 et seq. (1949).

43
Muhtar Çokar, Kürtaj 227 (2008).

44 1. Menstrual regulation (Mr) and other abortion kits. 2. Autoclave and electric sterilizer. 3. Uterotonics, hemostatic drugs, intravenous fluid set, injector, sterile gloves, and other medical tools and equipment. 4. Beds for patients to rest on after hysterectomy. 5. Electric or nonelectric vacuum aspirator.

45
For the current 2021 list, see: *WHO Model List of Essential Medicines—22nd list*
https://www.who.int/publications/i/item/WHO-MHP-HPS-EML-2021.02 (accessed Sept. 28, 2022), 50.

46 Laura Britton and Amy Bryant, *When Off-label Is Illegal: Implications of Mandating the FDA-approved Protocol for Mifepristone-induced Abortions*, 25 Women’s Health Issues 433 (2015).

47 Mariana Prandini Assis and Joanna N. Erdman, *In the name of public health: misoprostol and the new criminalization of abortion in Brazil*, 8 Journal of Law and the Bıoscıences lsab009, 14 (2021).

48 According to Article 152 of the General Hygiene Law: ‘The import and sale of tools and equipment that are instrumental in abortion and to be determined by the Ministry of Health and Internal Medicine are prohibited. All of these in medicine are imported under the conditions to be determined by the Ministry of Health and Social Aid and sold in pharmacies with a prescription’. Meanwhile, Ppl Art. 3, Para. 3 includes the following provision: ‘The quality of medicines and tools to be used in population planning shall be determined by the Ministry of Health and Social Welfare, with the written opinion of a commission including faculty members from medical faculties. Medicines and tools not determined in this way by the Ministry of Health and Social Assistance cannot be used on people by any unit, institution or organization, including medical faculties of universities’.

49 Elizabeth G. Raymond et al., *First-trimester medical abortion with mifepristone 200 mg and misoprostol: a systematic review*, 87 Contraceptıon 26, 32 (2013).

50 Işık Gönenç and Zeynep Tuzcular Vural, *Medical Abortion: An overview and management of Mifepristone related side effects and complications*, 1 The Journal of Turkısh Famıly Physıcıan 92, 93 (2010).

51 Michelle Oberman, *What will and won’t happen when abortion is banned*, 9 Journal of Law and the Bıoscıences lsac011, 7 (2022).

52 Halil Kaya et al., *Oral and Vaginal Misoprostol Use for Medical Abortion*, 2 Journal of Emergency Medıcıne Case Report 32, 34 (2011).

53 A. Causes related to obstetrics and gynecology 1. Previous major uterine injury and damage [(a) cesarean section, (b) myomectomy, (c) uterine rupture, (d) extensive perforation, and (e) previous vaginal plastic operations] 2. Recurrent preeclampsia or eclampsia 3. Isoimmunization 4. Mol hydatidiform B. Orthopedic causes 1. Osteogenesis imperfecta 2. Severe kyphoscoliosis 3. Osteomyelitis complicating birth 4. Osteoarticular diseases involving all joints in action C. Causes related to blood diseases 1. Leukemia 2. Diseases that cause chronic anemia 3. Lymphomas 4. Coagulation defects 5. Hemolytic jaundice 6. Agranulocytosis 7. Thromboembolic diseases 8. Hemoglobinopathies and thalassemia syndromes (causing severe clinical and hematologic impairment) 9. Gammopathy D. Diseases of the heart and circulatory system 1. Congenital and acquired heart diseases that prevent delivery 2. Heart failure, pericarditis, myocarditis, myocardial infarction, overt coronary insufficiency, aneurysms of the arterial system 3. Severe thrombophlebitis and lymphatic system diseases 4. Severe bronchiectasis 5. Chronic lung diseases that impair respiratory function E. Kidney diseases 1. Acute and chronic kidney diseases F. Eye diseases 1. Detachment 2. Renal hypertensive and diabetic retinopathies G. Endocrine and metabolic diseases 1. Pheochromocytoma 2. Adrenal hyperfunction or insufficiency 3. Uncontrolled hypothyroidism or hyperthyroidism 4. Parathyroid hyperfunction or insufficiency 5. Severe pituitary diseases H. Causes related to the digestive system 1. Diseases of the digestive organs that prevent the continuation of pregnancy I. Immunological causes 1. Immunodeficiency diseases 2. Collagen tissue diseases J. All malignant neoplastic diseases K. Neurological causes 1. Grand mal epilepsy 2. Multiple sclerosis 3. Muscular dystrophy 4. Hemiplegia and paraplegia 5. Severe neurological diseases that prevent the continuation of pregnancy L. Causes related to mental illness 1. Oligofreni 2. Chronic schizophrenia 3. Psychotic manic depressive (PMD) 4. Paranoia 5. Drug addictions and chronic alcoholism M. Infectious diseases 1. Teratogenic intrauterine infections [(a) rubella, (b) toxoplasmosis, (c) cytomegalovirus, and (d) herpes virus group diseases] 2. Leprosy 3. Malaria 4. Syphilis 5. Brucella and other severe chronic infections N. Congenital causes 1. Marfan syndrome 2. Bladder exstrophy 3. Down syndrome 4. Other hereditary diseases with a high probability of producing a defective child 5. X-rays and drugs harmful to the gonads 6. Teratogenic drugs 7. Neurofibromatosis

54 Aytül Çorbacıoğlu Esmer and Atıl Yüksel, *Termination of Pregnancies After Prenatal Diagnosis and Legal Status*, 10 Turkısh Journal of Publıc Health 83, 89 (2012).

55
Lisette Rodriguez-Cabezas and Crystal Clark, *Psychiatric Emergencies in Pregnancy and Postpartum*, 61 Clınıcal Obstetrıcs and Gynecology 615, 615–6 (2018).

56 Compare: Işıl Güney Tunalı, Hekimin Tıbbi Müdahalede Bulunma Yükümlülüğünün Sınırları 449 (2020).

57 Also see: Yener Ünver, *Türk Ceza Kanunu Açısından Çocuk Düşürtme ve Düşürme Suçları*, 6 The Journal of Yeditepe Unıversıty Faculty of Law 173, 191 et seq. (2009).

58 Çorbacıoğlu Esmer and Yüksel, *supra* note 54, at 89–90.

59 Compare: Büyükay, *supra* note 5, at 24.

60 Rosamund Scott, *The Uncertain Scope of Reproductive Autonomy in Preimplantation Genetic Diagnosis and Selective Abortion*, 13 Medıcal Law Revıew 291, 300 et seq. (2005).

61 For an example see: Çorbacıoğlu Esmer and Yüksel, *supra* note 54, at 90–1.

62 İsmail Dölen and Özlem Özdeğirmenci, *Viabilite Altı Gebeliklerin Sonlandırılması ve Etik*, 2 Turkısh Journal of Obstetrıcs and Gynecology 91, 92 (2003).

63
Sedef Erkmen, Türkiye’de Kürtaj: Akp ve Biyopolitika 103 (2020).

64 See: Elizabeth Chloe Romains, *Is ‘viability’ viable? Abortion, conceptual confusion and the law in England and Wales and the United States*, 7 Journal of Law and the Bıoscıences lsaa059, 21 et seq. (2020); Pap Claudia Mangel, *Legal Abortion: The Impending Obsolescence of The Trimester Framework*, 14 American Journal of Law & Medicine 69, 88 et seq. (1988).

65 Leo Han, Maria I. Rodriguez and Aaron B. Caughey, *Blurred Lines: Disentangling the Concept of Fetal Viability from Abortion Law*, 28 Women’s Health Issues 287 (2018).

66
Hakan Hakerİ, Tip Hukuku 728 (2012). Compare: Jale Akİpek, Turgut Akintürk and Derya Ateş, Türk Medenİ Hukuku 1. Cİlt: Başlangiç Hükümlerİ—Kİşİler Hukuku 370 (2021).

67 ‘The intervention specified in Article 5 depends on the permission of the pregnant woman, the consent of the minor with the consent of the guardian, and the permission of the magistrate with the consent of the minor and the guardian in persons who are under guardianship but are of minor age or incapacity. However, in the case of a pregnant woman who does not have the freedom of consciousness due to mental disability, her consent is not sought for the evacuation of the uterus’.

68 ‘…If persons mentioned in the first paragraphs of Article 5 and whose consent is sought are married…the consent of a spouse is also required for the evacuation of a uterus’.

69 1. Operating room and anesthesia tools and equipment 2. Medical and surgical equipment needed to perform a cesarean section 3. Necessary equipment for intra-amniotic hypertonic solution administration 4. Abortion kit 5. Electric or nonelectric vacuum aspirator 6. Autoclave and electric sterilizer 7. Uterotonics, hemostatic drugs, intravenous fluid set, injector, sterile gloves, etc. other medical tools and equipment 8. Anesthesiologist or technician 9. Other necessary operating room personnel 10. Resuscitation tools and equipment

70 According to the Regulation on Medical Deontology (RMD), abortion is possible when it is the only remedy to save the life of a pregnant person (Art. 22, Para 1, Sentence 1). In this case, the written consent of a patient and, if any, the parent or guardian is required (Art. 22, Para. 4).

71 M. Kemal Oğuzman, Özer Selİçİ and Saİbe Oktay Özdemİr, Kİşİler Hukuku 238–9 (2021).

72 Also see: Köksal Bayraktar, Hekİmİn Tedavİ Nedenİyle Cezaî Sorumluluğu 107 (1972).

73 In the same view on criminal liability: Dilek Özge Erdem, *Çocuk Düşürtme Düzenlemesi*, 65 Ankara Ünıversİtesİ Hukuk Fakültesİ Dergİsİ 1637, 1669 (2016). Opposing view: Meral Ekİcİ Şahİn, Ceza Hukukunda Riza 278 et seq., 312 et seq. (2012).

74
Oğuzman, Selİçİ and Oktay Özdemİr, *Supra* note 71, at 238.

75 Also see: İsmail Dölen, *Kürtaj Gerçeği - Türk Jinekoloji ve Obstetrik Derneği Görüşü*, 10 Turkish Journal of Public Health 75, 79–80 (2012).

76
Velİ Özer Özbek, Koray Doğan and Pinar Bacaksiz, Türk Ceza Hukuku Özel Hükümler 332 (2022).

77 In a similar opinion: Barış Işık, *Mağduru Olduğu Bir Suç   Sonucu Gebe Kalan Kadının Çocuğunun Düşürtülmesi Suçu (TCK m. 99/6)*, 3 Journal of Penal Law and Criminology 25, 39 (2015). Compare: Durmuş Tezcan, Mustafa Ruhan Erdem and Murat Önok, Teorİk ve Pratİk Ceza Özel Hukuku 334 fn. 78 (2022).

78
Tcc Art. 129 includes the provision ‘Marriage is forbidden between the following persons: 1. Between lineal descendants, between siblings, between uncles, aunts and nephews, nieces; 2. Between one of the spouses and the other’s lineal descendant, even if the marriage which gave rise to the kinship of in-laws has ended; 3. Between the adopter and the adopted child or between one of them and the other’s descendants and spouse’.

79 Sibel Can, *Çocuk Düşürtme Suçunun Suç  Sonucu Oluşan Gebelikler Açısından Değerlendirilmesi*, 73 Ankara Bar Revıew 79, 97 (2015).

80 See: Center for Reproductive Rights, *supra* note 9. For example: Poland’s The Family Planning, Human Embryo Protection and Conditions of Permissibility of Abortion Act Art. 4a, Para. 1, Sub-Para. (3). In Polish law, such relationships are also criminalized (Poland Criminal Code Art. 201).

81 See: Center for Reproductive Rights, *supra* note 9. For example: Federal Law on the Basics of Health Protection of the Citizens in the Russian Federation Art. 56 and Government Decree on the Social Indication for the Artificial Termination of Pregnancy.

82 Serap Keskin Kiziroğlu, *Çocuk Düşürme ve Düşürtme Suçları*, in Özel Ceza Hukuku Cİlt Ii: Kİşİlere Karşi Suçlar 446 (2017).

83 ‘(1) In order to obtain evidence of a crime, it is possible to conduct an external or internal body examination on the body of the victim, or to take blood or similar biological samples, such as hair, saliva, fingernails; provided that they do not endanger his health and do not involve any surgical intervention; it may be decided by the judge or court upon the request of the public prosecutor or ex officio, or by the public prosecutor in cases of inconvenience in delay. The decision of the public prosecutor is submitted to the approval of the judge or court within twenty-four hours. The judge or court gives its decision within twenty-four hours. Decisions that are not approved remain null and void and evidence obtained cannot be used. (2) If the victim has consented, there is no need to take a decision in accordance with the first paragraph in order to carry out these actions. (3) In case it is necessary to investigate the paternity of the child, a decision pursuant to the provision of the first paragraph must be taken in order to conduct this investigation. (4) Examination or taking samples from the body may be avoided for reasons of refraining from witnessing. The legal representative decides on the abstention of children and the mentally ill. If the child or the mentally ill person is in a position to perceive the legal meaning and consequences of the testimony, his/her opinion is also taken. If the legal representative is also a suspect or accused, the judge decides on this issue. However, the evidence obtained in this case cannot be used in the further stages of the case without the consent of the legal representative who is not the suspect or the accused. (5) Decisions of a judge or court made pursuant to this article may be appealed’.

84 ‘In cases where a person suspected of committing or participating in a crime is believed to have possible evidence of the crime committed by him or her in his or her body or in the body of the victim, it is subject to the decision of the judge to subject the accused or the victim to medical treatment in order to reveal this evidence. In cases where delay is inconvenient, this action can be carried out upon the request of the public prosecutor’.

85
Walter Gropp and Liane Wörner, in Münchener Kommentar Zum Strafgesetzbuch: Stgb, Volume 4: §§ 185-262 1007 (Volker Erb and Jürgen Schäfer eds. 2021).

86 Berrin Akbulut and Ola Raheem Kareem, *Türk Ceza Kanunu ve Irak Ceza Kanununda Çocuk Düşürtme ve Düşürme Suçları*, 13 Jornal of Criminal Law 11, 51 (2018).

87 Ole Ingemann-Hansen and Annie Vesterby Charles, *Forensic medical examination of adolescent and adult victims of sexual violence*, 27 Best Practice & Research Clinical Obstetrics and Gynaecology 91, 95–6 (2013).

88
*R.G. App.* (App. 2017/31619).

89 According to the TCC, although puberty is acquired upon reaching the age of 18, marriage renders a person an adult (Art. 11). Neither a man nor a woman may marry until they are 17 years of age (Art. 124, Para. 1). A child over the age of 17 may marry with the consent of their legal representative (Art. 126). A child over the age of 16 may marry only in extraordinary circumstances and with the permission of a judge for a very important reason. Before making a decision, the judge must, if possible, hear the parents or guardian of the child (Art. 124). It is clear, however, that these legal regulations permitting child marriage are inaccurate and should be eliminated as soon as possible.

90 A minor who has reached the age of 15 may be rendered an adult by the court upon their own request and with the consent of their parents (TCC Art. 12).

91 For more information, see: Mehmet Nihat Kanbur, *6545 Sayılı Kanun Değişiklikleri Çerçevesinde Türk Ceza Kanunu’nda Reşit Olmayanla Cinsel İlişki Suçu (Tck m.104)*, 16 Dokuz Eylul University Faculty of Law Review 4139, 4161 et seq. (2014).

92 Turkish Court of Appeals for the 14th circuit, Merits No: 2011/12610, Decision No: 2012/460 (Jan. 19, 2012).

93 For more information, see: Ali Tanju Sarıgül, *Reşit Olmayanla Cinsel İlişki Suçunda Fail (Tck m. 104)*, 25 Ankara Haci Bayram Veli University Faculty of Law Review 711, 735 et seq. (2021).

94 As an example among many decisions: Turkish Court of Appeals for the 14th circuit, Merits No: 2018/8581, Decision No: 2020/2772 (June 29, 2020).

95 Turkish court of appeals for the 14th circuit, Merits No: 2012/13961, Decision No: 2013/467 (Jan. 24, 2013).

96
Heidi K. Leftwich and Marcus Vinicius Ortega Alves, *Adolescent Pregnancy*, 64 Pediatric Clinics of North America 381, 383 (2017).

97 Because of the presumption of paternity, the father of the child born by a pregnant person as a result of a crime in which the perpetrator is not her spouse or former spouse while she is married or within 300 days starting from the end of the marriage is the spouse or former spouse. However, the action for denial of paternity (TCC Art. 286 et seq.) can be opened to rebut this presumption.

98 Also see: Burcu Dönmez, *TCK’da Çocuk Düşürme Suçu Mukayeseli Hukuk ve AİHM Bakış Açısıyla Ceninin Yaşama Hakkının Sınırlandırılması*, 9 Dokuz Eylul University Faculty of Law Review 99, 135–6 (2007).

99
https://gender.khas.edu.tr/sites/gender.khas.edu.tr/files/docs/2020-12/2020-kurtaj-arastirmasi-raporu.pdf (accessed Sept. 12, 2022).

100
https://gender.khas.edu.tr/sites/gender.khas.edu.tr/files/docs/2020-12/2020-kurtaj-arastirmasi-raporu.pdf (accessed Sept. 12, 2022).

101 Greer Donley, Beatrice A. Chen and Sonya Borrero, *The legal and medical necessity of abortion care amid the COVID-19 pandemic*, 7 Journal of Law and the Biosciences lsaa013, 10 et seq. (2020).

102 Also see: Iqbal H. Shah, *Safe abortion services: a reproductive health imperative*, 10 Turkish Journal of Public Health 10, 12 et seq. (2012); Heidi Moseson et al., *Contextualizing medication abortion in seven African nations: A literature review*, 40 Health Care For Women International 950, 954 et seq. (2019).

103
*Supra* note 32, at 15.

104 Özel, Engin Üstün and Avşar, *Supra* note 16, at 35.

105 For example: Holland Law on The Termination of Pregnancy Art. 20.

106
*R.R. v Poland* (App. 27617/04).

107 See: Elizabeth Chloe Romains, Alexandra Mullock and Jordan A. Parsons, *The Excessive Regulation of Early Abortion Medication in the UK: The Case for Reform*, 30 Medical Law Review 4, 16 et seq. (2021).

